# A “stepped” flexible vacuum-assisted ureteral access sheath improves stone clearance efficiency: experimental results from an *ex vivo* porcine model

**DOI:** 10.3389/fsurg.2026.1959554

**Published:** 2026-09-30

**Authors:** Daibing Lv, Ming Jin, Xi Meng, Weizhong Zhang, Shanfang Yuan, Yang Ding, Weixi Wang, Jianghua Yang, Xiaoxing Liao

**Affiliations:** Department of Urology, Beijing University of Chinese Medicine Third Affiliated Hospital, Beijing, China

**Keywords:** "Stepped" flexible vacuum-assisted ureteral access sheath, *ex vivo* model, irrigation flow rate, stone clearance efficiency, ureteroscopy

## Abstract

**Objective:**

To evaluate the impact of a novel “stepped” flexible vacuum-assisted ureteral access sheath (SFV-UAS) on stone clearance efficiency during flexible ureteroscopy lithotripsy (f-URL).

**Methods:**

Human upper urinary tract stone fragments (< 4 mm) were placed into *ex vivo* porcine renal pelves. We simulated f-URL stone extraction under continuous irrigation and vacuum suction, comparing the SFV-UAS (ureteral segment 12/14 Fr, cystourethral segment 16/18 Fr) with a conventional flexible vacuum-assisted ureteral access sheath (FV-UAS)(12/14 Fr). With intrarenal pressure strictly controlled (< 30 mmHg) and constant vacuum suction (01 step), irrigation flow rates were evaluated at 40 mL/min, 50 mL/min, 60 mL/min, 70 mL/min, 80 mL/min, and 90 mL/min. Stone evacuation times were recorded.

**Results:**

(1) Sheath type and irrigation flow rate exhibited significant main effects on clearance time [F(1,408) = 189.231, *P* < 0.01, effect size 31.7%; and F(5,408) = 38.721, *P* < 0.01, effect size 32.2%, respectively]. A significant two-way interaction was also observed [F(5,408) = 8.372, *P* < 0.01, effect size 9.3%]. (2) Simple main effects revealed: First, across all corresponding irrigation rates, the SFV-UAS group achieved significantly shorter clearance times than the FV-UAS group (mean differences: MD = −21.598, −18.099, −8.172, −11.129, −9.762, −4.883; all *P* < 0.05). Second, within the SFV-UAS group, rates ≥ 70 mL/min resulted in significantly shorter clearance times vs. the 40 mL/min baseline (mean differences:MD = −9.393, −9.771, −9.084; all *P* < 0.05). Although comparisons between 60 mL/min and higher rates were non-significant, 70 mL/min was the lowest evaluated irrigation rate that demonstrated a statistically significant reduction in clearance time compared to the 40 mL/min baseline. Third, within the FV-UAS group, clearance times at ≥ 60 mL/min were significantly shorter than at 40 and 50 mL/min (mean differences:MD = −19.664, −12.370; all *P* < 0.001), with no significant differences among the ≥ 60 mL/min groups, indicating its plateau at ≥ 60 mL/min.

**Conclusion:**

Within the parameters of this *ex vivo* model,the SFV-UAS significantly reduces the measured stone clearance time during f-URL compared to the FV-UAS under identical irrigation conditions. For the SFV-UAS group, targeting an irrigation flow rate of ≥ 70 mL/min consistently optimizes stone extraction efficiency relative to baseline conditions.

## 1 Introduction

Flexible ureteroscopy lithotripsy (FURL) is a common treatment for upper urinary tract calculi. With continuous advancements in surgical techniques and related instrumental equipment, its clinical indications are gradually expanding. In recent years, the application of the flexible vacuum-assisted ureteral access sheath (FV-UAS) during FURL has successfully integrated lithotripsy with active stone aspiration, leading to higher postoperative stone-free rates (1). A critical step in this procedure is the evacuation of stone fragments, specifically those smaller than the diameter of ureteral access sheath (UAS) but larger than the flexible endoscope body. This extraction process requires the precise coordination of three key elements: the irrigation flow rate, the vacuum suction pressure, and the withdrawal speed of the flexible endoscope. Currently, the stone evacuation path from the renal pelvis or calyces via scope withdrawal is approximately 50 cm long (equivalent to the standard length of an FV-UAS), which renders each extraction cycle significantly time-consuming. To address this technical bottleneck, we designed and patented a novel “stepped” flexible vacuum-assisted ureteral access sheath (SFV-UAS; Patent No. ZL 2024 2 1600735.3) based on the anatomy of the human urinary tract. This innovative design successfully shortens the physical path of stone evacuation to merely the length of the ureter. To evaluate the differences in intraoperative stone clearance efficiency between the novel SFV-UAS and the conventional FV-UAS, we conducted a comparative experimental study using an *ex vivo* porcine FURL model. The findings are reported herein.

## 2 Materials and methods

### 2.1 Materials

SFV-UAS (12/14 Fr; Zhejiang Zhexin Co., Ltd., Zhejiang, China) (Figures 1Aa).

FV-UAS (12/14 Fr; Reborn Medical, Zhejiang, China) (Figures 1Ab).

Percutaneous renal access sheath (12/14 Fr; Laike Medical Devices, Beijing, China) (Figure 1B).

**Figure 1:**
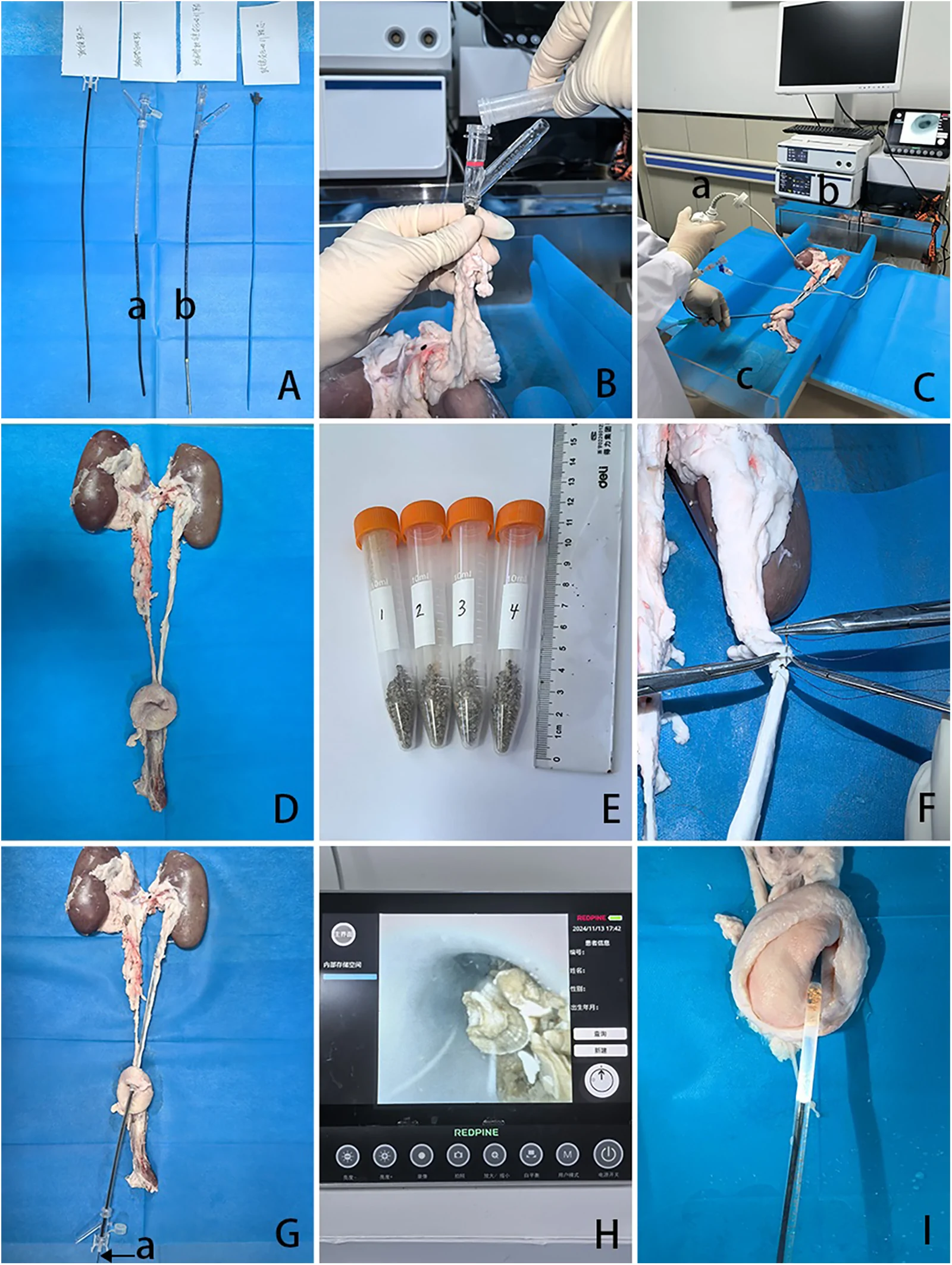
**(A)** Two types of ureteral access sheaths and their obturators: **(a)** SFV-UAS, with the lower section being the ureteral segment and the upper section being the cystourethral segment; **(b)** FV-UAS. **(B)** Instillation of stone fragments into the porcine renal pelvis through a 12/14 Fr percutaneous renal access sheath. **(C)** Illustration of the FURL procedure under vacuum suction: **(a)** single-use digital flexible ureteroscope; **(b)** medical pressure-controlled suction system; **(c)** container equipped with a high-transparency acrylic plate. **(D)** Intact porcine urinary tract organs (male): kidneys, ureters, bladder, and urethra. **(E)** Human renal stone fragments (< 4 mm in diameter). **(F)** Closure of the ureteral incision using a 3−0 suture. **(G)** Placement of the SFV-UAS over a hydrophilic guidewire: **(a)** hydrophilic guidewire. **(H)** Endoscopic view of intrarenal stones under the flexible ureteroscope. **(I)** During the procedure in the SFV-UAS group, stone fragments are evacuated to the wedge-shaped junction of the cystourethral segment of the SFV-UAS.

Single-use digital flexible ureteroscope (8.5 Fr; Redpine, Guangdong, China) (Figures 1Ca).

Pressure-controlled medical suction system (Puyue Medical, Shanghai, China) (Figures 1Cb).

Reservoir tank made of high-transparency acrylic board (Figures 1Cc).

Intact fresh porcine urinary tract system, comprising the kidneys, ureters, bladder, and urethra (Figure 1D).

Human upper urinary tract stone fragments (Figure 1E) (To ensure morphological and mass standardization, the naturally irregular fragments were meticulously passed through standard test sieves to isolate a uniform size < 4 mm. This strict sieving process guaranteed that the individual fragments targeted during each extraction cycle possessed comparable physical mass and aerodynamic profiles).

Surgical suture (3–0; Corza Medical, Shanghai, China) (Figure 1F).

Hydrophilic guidewire (0.33 mm in diameter; Huamei Medical, Shanghai, China) (Figure 1Ga).

Medical endoscopic image processor (Redpine, Guangdong, China) (Figure 1H).

### 2.2 Experimental procedure

The intact fresh porcine urinary tract system (comprising the kidneys, ureters, bladder, and urethra) (Figure 1D) was placed in a container made of a high-transparency acrylic plate (Figures 1Cc), and the adipose tissue surrounding the kidneys, ureters, and bladder was removed. Based on the position of the internal urethral orifice, the ureteral orifices were located (the anterior wall of the bladder had been incised), a hydrophilic guidewire was advanced into the renal pelvis, and an incision was made on the ureter approximately 5 cm from the renal pelvis. A 12/14 Fr "Y"-shaped percutaneous renal access sheath was inserted into the renal pelvis and maintained in a vertical position. Subsequently, an initial total volume of 2 cm^3^ of human upper urinary tract stone fragments was instilled into each kidney to establish a continuous stone reservoir, simulating a significant stone burden (Figures 1B,E). The ureteral incision was closed using a 3–0 suture to simulate the state of human upper urinary tract calculi for subsequent use (Figure1F).

The SFV-UAS (Figures 1Aa) was advanced into the renal pelvis along the hydrophilic guidewire. After withdrawing the guidewire, a single-use digital flexible ureteroscope (Figures 1Ca) was inserted for observation (Figure 1H). A medical pressure-controlled suction system (including an automated irrigation system and an automated vacuum suction system) was connected (Figures 1Cb). The actual intrarenal pressure (IRP) was continuously monitored in real-time via a dedicated pressure sensor integrated into the distal tip of the flexible ureteroscope, strictly maintaining the IRP ≤ 30 mmHg. The vacuum suction was operated in “01 step”(Mode 1, Set the actual IRP to ≤30 mmHg), and the irrigation flow rates were set to 40 mL/min, 50 mL/min, 60 mL/min, 70 mL/min, 80 mL/min, and 90 mL/min, respectively. In the SFV-UAS group, the stone fragments were evacuated from the renal pelvis to the wedge-shaped junction of the stepped sheath by withdrawing the flexible endoscope during each maneuver. This clearance process was confirmed in real-time through two methods: macroscopically, via the transparent structure of the cystourethral segment of the sheath (Figures 1Aa), and internally, under direct endoscopic visualization as the fragments entered the widened cystourethral segment (Figure 1I). During each scope withdrawal cycle, single or multiple stone fragments (<4 mm) were cleared to ensure procedural reproducibility, rather than utilizing a specific mass or weight as the extraction standard. The operation time for each withdrawal maneuver was recorded. Considering the short duration of each operation, this maneuver was repeated 35 times under the same flexible sheath and irrigation speed conditions. All 420 stone extraction trials were performed by a single experienced urologist who has completed over 1,000 flexible ureteroscopy procedures. Auxiliary tasks, such as time recording, were consistently managed by dedicated personnel. The sequence of trials under each experimental condition was randomized. Furthermore, adequate rest intervals were incorporated between maneuvers to minimize operator fatigue and mitigate any potential bias from the learning curve. To prevent tissue degradation from biasing the outcomes, the 420 extraction trials were distributed across four independent *ex vivo* kidney-ureter units. Macroscopic tissue integrity was continuously monitored, with no evidence of ureteral perforation or significant mucosal avulsion observed. More importantly, the strict randomization of the testing sequence ensured that any subtle, time-dependent biomechanical alterations in the ureteral wall were equally distributed across all experimental groups, thereby negating systemic bias.

During the FURL procedure in the conventional sheath group, the FV-UAS was used instead. The distance for each stone extraction via scope withdrawal was to completely clear the stone fragments from the renal pelvis to the distal end of the FV-UAS (Figures 1Ga). All other procedures were identical to those in the stepped sheath group.

### 2.3 Statistical analysis

All data were analyzed using SPSS 20.0 statistical software (IBM Corp., Armonk, NY, USA). All measurement data were expressed as the mean ± standard deviation and computed using a multifactorial analysis of variance (General Linear Model - Univariate), with the data successfully passing the homogeneity of variance test. Descriptive statistical analysis was initially performed, followed by Levene's test for equality of error variances to obtain the between-subjects effects. Based on the between-subjects effects, it was determined whether a further interaction effect test between the two factors was required. Finally, the simple effect results for the different sheath type groups and different irrigation flow rate groups were obtained through *post hoc* comparisons.

## 3 Results

During the FURL procedure in the SFV-UAS group, utilizing the extraction maneuver by withdrawing the endoscope, stone fragments could be evacuated from the renal pelvis to the wedge-shaped junction at the cystourethral segment of the stepped sheath; in the FV-UAS group, stone fragments could be evacuated from the renal pelvis to the distal end of the flexible ureteral access sheath. A total of 420 data points for stone extraction time were obtained in this experiment. Descriptive statistical analysis yielded the values of the time required for stone extraction at each level of each factor (Table 1).

**Table 1:** Descriptive analysis of stone retrieval time by flexible ureteral access sheath and irrigation rate groups.

Access sheath group	Irrigation rate group (mL/min)	Mean (s)	Standard deviation	N
SFV-UAS	40	16.8186	5.69950	35
	50	13.0234	3.16350	35
	60	10.5803	4.02096	35
	70	7.4254	1.87013	35
	80	7.0480	2.12164	35
	90	7.7349	1.57734	35
	Total	10.4384	4.88440	210
FV-UAS	40	38.4169	19.18789	35
	50	31.1223	8.13151	35
	60	18.7526	12.32135	35
	70	18.5540	6.27719	35
	80	16.8103	15.06383	35
	90	12.6177	9.02818	35
	Total	22.7123	15.28068	210
Total	40	27.6177	17.76902	70
	50	22.0729	10.98144	70
	60	14.6664	9.98565	70
	70	12.9897	7.24909	70
	80	11.9291	11.75602	70
	90	10.1763	6.88740	70
	Total	16.5754	12.88887	420

Based on the tests of between-subjects effects, the following conclusions can be drawn: 1. The main effect of different types of UAS groups on stone clearance time was significant [F(1, 408) = 189.231, *P* < 0.01], indicating that different types of UAS significantly affected stone clearance time, with an effect size of 31.7%; 2. The main effect of different irrigation flow rate groups on stone clearance time was significant [F(5, 408) = 38.721, *P* < 0.01], indicating that different irrigation flow rates significantly affected stone clearance time, with an effect size of 32.2%; 3. A pairwise interaction existed between the different types of UAS and different irrigation flow rates, and this second-order effect had a significant impact on stone clearance time [F(5, 408) = 8.372, *P* < 0.01], indicating that the combination of different UAS types and irrigation flow rates significantly affected the clearance time, with an effect size of 9.3%, which was lower than the main effects of the individual factors (Table 2). The trends of the interaction effect between the two factors on stone extraction time are illustrated in Figure 2A.

**Table 2:** The table of inter-subject effects.

The source	Type III sum of squares	Degree of freedom	Mean square	F	Significance	Partial eta squared
Calibration model	35,500.502a	11	3,227.318	38.609	.000	.510
intercept	115,391.835	1	115,391.835	1,380.438	.000	.772
Different types of sheaths	15,817.995	1	15,817.995	189.231	.000	.317
Different types of perfusion flow rate	16,183.470	5	3,236.694	38.721	.000	.322
Different types of sheaths* Different types of perfusion flow rate	3,499.038	5	699.808	8.372	.000	.093
Error	34,105.032	408	83.591			
Total	184,997.370	420				
Total corrected	69,605.535	419				
a. R Square = .510（ Adjust R square = .497）						

**Figure 2:**
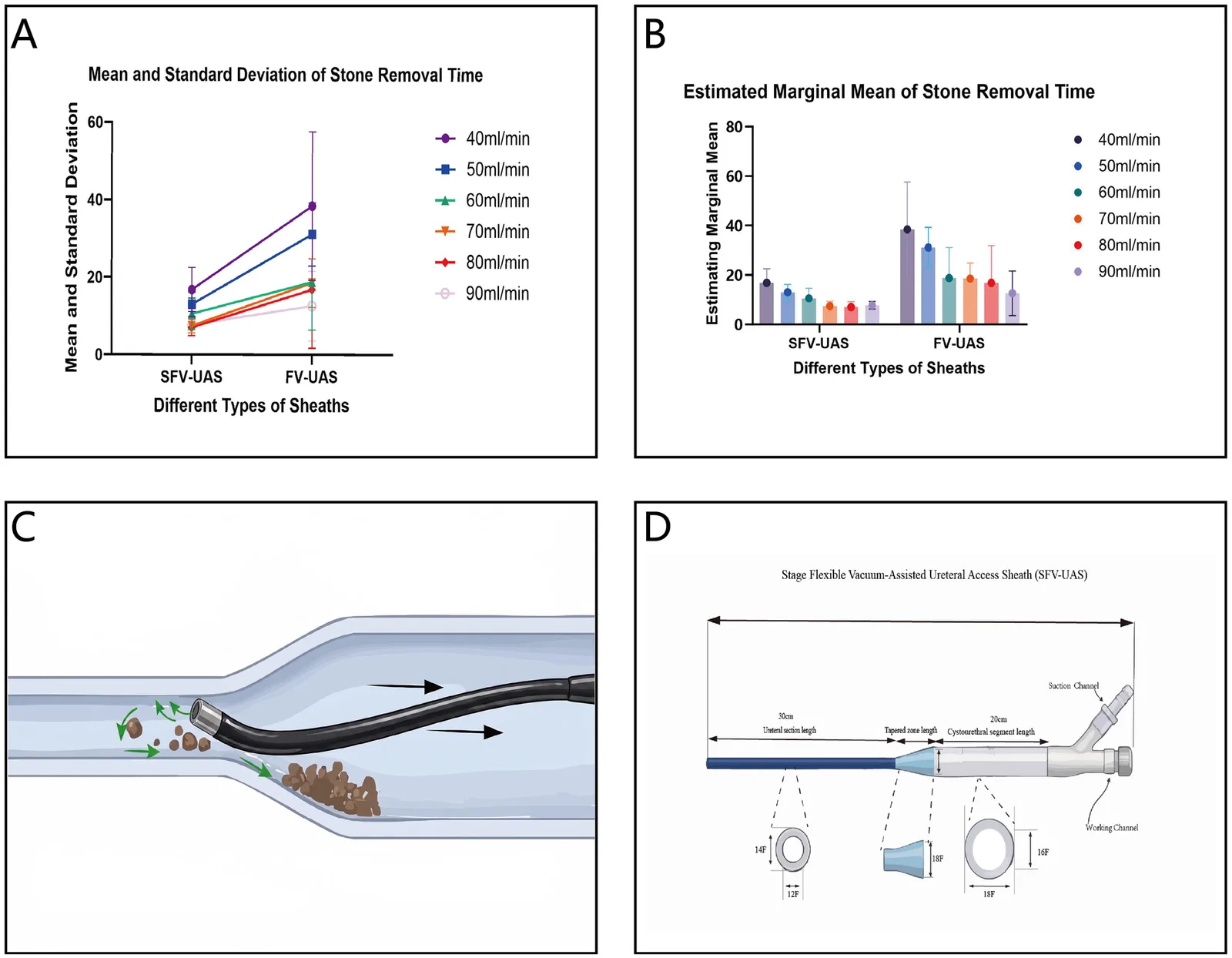
**(A)** line chart illustrating the mean and standard deviation of stone extraction time across different sheath types. **(B)** Line chart displaying the estimated marginal means of stone extraction time across different sheath types. **(C)** Schematic diagram demonstrating stone fragments being aspirated into the ureteral segment of the SFV-UAS under negative pressure, with green arrows indicating the fluid vortices. **(D)** Structural schematic of the Stepped flexible vacuum-assisted ureteral access sheath (SFV-UAS).

Based on the simple effects results of stone extraction time for different flow rates and different sheath types, differences in the variance of extraction time among patients with different irrigation speeds and flexible sheaths were indicated: at irrigation rates of 40 mL/min, 50 mL/min, 60 mL/min, 70 mL/min, 80 mL/min, and 90 mL/min, the stone extraction time in the stepped sheath group was significantly shorter than that in the conventional sheath group (MD = −21.598, *P* < 0.001; F = −18.099, *P* < 0.001; MD = −8.172, *P* < 0.001; MD = −11.129, *P* < 0.001; MD = −9.762, *P* < 0.001; MD = −4.883, *P* = 0.026 < 0.05) (Table 3). The estimated marginal means of stone extraction time for the two groups at each irrigation flow rate are visually presented in Figure 2B.

**Table 3:** Simple effect results for different perfusion rates with different types of sheaths.

Irrigation rate group (mL/min)	(I) Sheath group	(J) Sheath group	Mean Difference (I-J)	Standard error	Significance^b^
40	SFV-UAS	FV-UAS	−21.598*	2.186	.000
	FV-UAS	SFV-UAS	21.598*	2.186	.000
50	SFV-UAS	FV-UAS	−18.099*	2.186	.000
	FV-UAS	SFV-UAS	18.099*	2.186	.000
60	SFV-UAS	FV-UAS	−8.172*	2.186	.000
	FV-UAS	SFV-UAS	8.172*	2.186	.000
70	SFV-UAS	FV-UAS	−11.129*	2.186	.000
	FV-UAS	SFV-UAS	11.129*	2.186	.000
80	SFV-UAS	FV-UAS	−9.762*	2.186	.000
	FV-UAS	SFV-UAS	9.762*	2.186	.000
90	SFV-UAS	FV-UAS	−4.883*	2.186	.026
	FV-UAS	SFV-UAS	4.883*	2.186	.026

Based on the different sheath types and irrigation flow rates, the following simple effects results can be obtained:

1. In the stepped sheath group, the stone extraction time at an irrigation rate of 40 mL/min was significantly longer than those at 70 mL/min, 80 mL/min, and 90 mL/min (MD = 9.393, *P* < 0.001; MD = 9.771, *P* < 0.001; MD = 9.084, *P* = 0.001 < 0.05);
2. In the stepped sheath group, the stone extraction time at 70 mL/min was significantly shorter than that at 40 mL/min (MD = −9.393, *P* < 0.001);
3. In the stepped sheath group, the stone extraction time at 80 mL/min was significantly shorter than that at 40 mL/min (MD = −9.771, *P* < 0.001);
4. In the stepped sheath group, the stone extraction time at 90 mL/min was significantly shorter than that at 40 mL/min (MD = −9.084, *P* = 0.001 < 0.05);
5. In the conventional sheath group, the stone extraction time at 40 mL/min was significantly longer than that at 50 mL/min (MD = 7.295, *P* = 0.014 < 0.05), and significantly longer than those at 60 mL/min, 70 mL/min, 80 mL/min, and 90 mL/min (MD = 7.295, *P* = 0.014 < 0.05;MD = 19.664, *P* < 0.001; F = 19.863, *P* < 0.001; MD = 21.607, *P* < 0.001;MD = 25.799, *P* < 0.001);
6. In the conventional sheath group, the stone extraction time at 50 mL/min was significantly shorter than that at 40 mL/min (MD = 7.295, *P* = 0.014 < 0.05), and significantly longer than those at 60 mL/min, 70 mL/min, 80 mL/min, and 90 mL/min (MD = 12.370, *P* < 0.001; MD = 12.568, *P* < 0.001; MD = 14.312, *P* < 0.001; MD = 18.505, *P* < 0.001);
7. In the conventional sheath group, the stone extraction time at 60 mL/min was significantly shorter than those at 40 mL/min and 50 mL/min (MD = −19.664, *P* < 0.001; MD = −12.370, *P* < 0.001);
8. In the conventional sheath group, the stone extraction time at 70 mL/min was significantly shorter than those at 40 mL/min and 50 mL/min (MD = −19.863, *P* < 0.001; MD = −12.568, *P* < 0.001);
9. In the conventional sheath group, the stone extraction time at 80 mL/min was significantly shorter than those at 40 mL/min and 50 mL/min (MD = −21.607, *P* < 0.001; MD = −14.312, *P* < 0.001);
10. In the conventional sheath group, the stone extraction time at 90 mL/min was significantly shorter than those at 40 mL/min and 50 mL/min (MD = −25.799, *P* < 0.001; MD = −18.505, *P* < 0.001) (Table 4).

**Table 4:** Simple effect results for different types of sheaths versus different perfusion rate.

Access sheath group	(I) Irrigation rate group (mL/min)	(J) Irrigation rate group (mL/min)	Difference of means(I-J)	Standard error	Significance^a^
SFV-UAS	40	50	3.795	2.186	.728
		60	6.238	2.186	.066
		70	9.393*	2.186	.000
		80	9.771*	2.186	.000
		90	9.084*	2.186	.001
	50	40	−3.795	2.186	.728
		60	2.443	2.186	.990
		70	5.598	2.186	.150
		80	5.975	2.186	.094
		90	5.289	2.186	.214
	60	40	−6.238	2.186	.066
		50	−2.443	2.186	.990
		70	3.155	2.186	.912
		80	3.532	2.186	.816
		90	2.845	2.186	.960
	70	40	−9.393*	2.186	.000
		50	−5.598	2.186	.150
		60	−3.155	2.186	.912
		80	.377	2.186	1.000
		90	-.309	2.186	1.000
	80	40	−9.771*	2.186	.000
		50	−5.975	2.186	.094
		60	−3.532	2.186	.816
		70	-.377	2.186	1.000
		90	-.687	2.186	1.000
	90	40	−9.084*	2.186	.001
		50	−5.289	2.186	.214
		60	−2.845	2.186	.960
		70	.309	2.186	1.000
		80	.687	2.186	1.000
FV-UAS	40	50	7.295*	2.186	.014
		60	19.664*	2.186	.000
		70	19.863*	2.186	.000
		80	21.607*	2.186	.000
		90	25.799*	2.186	.000
	50	40	−7.295*	2.186	.014
		60	12.370*	2.186	.000
		70	12.568*	2.186	.000
		80	14.312*	2.186	.000
		90	18.505*	2.186	.000
	60	40	−19.664*	2.186	.000
		50	−12.370*	2.186	.000
		70	.199	2.186	1.000
		80	1.942	2.186	.999
		90	6.135	2.186	.076
	70	40	−19.863*	2.186	.000
		50	−12.568*	2.186	.000
		60	-.199	2.186	1.000
		80	1.744	2.186	1.000
		90	5.936	2.186	.098
	80	40	−21.607*	2.186	.000
		50	−14.312*	2.186	.000
		60	−1.942	2.186	.999
		70	−1.744	2.186	1.000
		90	4.193	2.186	.577
	90	40	−25.799*	2.186	.000
		50	−18.505*	2.186	.000
		60	−6.135	2.186	.076
		70	−5.936	2.186	.098
		80	−4.193	2.186	.577

a Adjustment for multiple comparisons: Bonferroni.

## 4 Discussion

In recent years, FURL has been widely adopted for the treatment of upper urinary tract calculi. Reducing intraoperative intrarenal pressure while concurrently enhancing the stone clearance rate has consistently remained a research focus for the optimization of this technique (2, 3). The flexible ureteral access sheath (UAS) is one of the most crucial auxiliary devices for FURL. Building upon the conventional UAS, the FV-UAS incorporates continuous vacuum suction and reaches the stone site at a close distance, demonstrating the advantage of simultaneous lithotripsy and active stone aspiration (4). Studies have shown that with the assistance of intelligent pressure control, the FV-UAS can prevent excessive renal function damage caused by elevated intraluminal pressure, reduce various adverse reactions resulting from surgical maneuvers, and promote patient postoperative recovery (1, 5, 6). However, during the clinical application of FV-UAS, when it is necessary to evacuate stone fragments larger than the gap between the inner diameter of the UAS and the outer diameter of the flexible endoscope, the conventional method involves withdrawing the endoscope aided by the vortex generated by irrigation and vacuum suction. Because the FV-UAS length is 50 cm or more (in males), the endoscope must undergo a relatively long withdrawal path. High-frequency repetitive scope withdrawal maneuvers not only prolong the operative time and increase surgeon fatigue, but also elevate the risk of patient complications (7), which is particularly evident when managing a large stone burden (8, 9). To address this clinical dilemma, this study evaluated, for the first time, the stone extraction efficiency of a novel “stepped” flexible vacuum-assisted ureteral access sheath (SFV-UAS) in a successfully established *ex vivo* porcine renal stone model, for which a patent has been successfully applied (Patent No. ZL 2024 2 1600735.3). The experiment revealed that the application of the stepped UAS shortened the scope withdrawal path during FURL, significantly reducing the stone extraction time compared with the FV-UAS.

The segmented diameter design of the SFV-UAS is fundamentally based on the anatomical differences of various segments of the human urinary tract. Due to the long and narrow anatomical characteristics and poor ischemia tolerance of the human ureter, the caliber of the UAS typically ranges from 11 to 14 Fr. Previous studies have confirmed that the use of a UAS larger than 12/14 Fr significantly increases the risk of ischemic injury to the ureteral wall and postoperative ureteral stricture (10). Therefore, in the design of our stepped UAS, the diameter of the ureteral segment remains identical to that of a conventional flexible access sheath. Based on anatomical characteristics, the adult urethra is wider in diameter than the ureter; in routine endourological surgeries, such as transurethral resection of the prostate (TURP) or transurethral resection of bladder tumors (TURBT), the outer sheath diameter of the resectoscope typically reaches 24 to 26 Fr. Considering the aforementioned anatomical factors, we designed the SFV-UAS with a ureteral segment diameter of 12/14 Fr and a cystourethral segment expanded to 16/18 Fr. According to Poiseuille's law, fluid resistance is inversely proportional to the fourth power of the pipe radius. Previous computational fluid dynamics studies have verified that during FURL, a minute increase in the sheath diameter can lead to a significant enhancement in outflow and an exponential reduction in resistance (11). When the fluid-stone mixture accelerates from the narrow ureteral segment into the wider cystourethral segment, flow separation occurs at the boundary due to the sudden increase in cross-sectional area, generating local fluid vortices (12). Upon reaching the wedge-shaped enlargement, the majority of stone fragments are automatically evacuated extracorporeally through the expanded cystourethral segment (16/18 Fr) driven by continuous irrigation and vacuum suction (Figure 2C). A minor proportion of residual fragments may temporarily lodge at the junction, which are subsequently cleared *en bloc* without the need for secondary evacuation maneuvers during the final withdrawal of the stepped sheath at the procedure's conclusion. In this way, the time required to complete a single stone extraction via scope withdrawal is naturally shortened to a withdrawal path equivalent to the length of the ureter. Studies have shown that the average length of the adult ureter is approximately 28 cm (13). Based on these anatomical data, the ureteral segment of the SFV-UAS is designed with a length of 30 cm, and the cystourethral segment has a length of 20 cm, connected by a 1-cm wedge-shaped structure. This design primarily facilitates the repetitive insertion and withdrawal of the flexible endoscope required during stone extraction maneuvers (Figure 2D). Furthermore, to address potential mechanical vulnerabilities such as kinking or mucosal shear during sheath insertion, this 1-cm tapered transition zone is engineered with a gradient flexibility profile. Combined with a continuous hydrophilic coating, this structural transition maintains mechanical integrity while minimizing insertion resistance and frictional trauma to the urothelium.

The present study found that during FURL utilizing the SFV-UAS, the stone extraction time was significantly shorter compared to the FV-UAS group. The experimental results indicated that under identical irrigation flow rates, the stone clearance time in the SFV-UAS group during FURL was significantly shorter than that in the FV-UAS group, with a significant main effect (MD = 189.231, *P* < 0.01) and an effect size of 31.7%. Although a pairwise interaction existed between the two factors of sheath type and irrigation flow rate, its effect size was 9.3%, which was lower than the main effects of the individual sheath type and irrigation flow rate groups. Our study further revealed that while the differences in extraction time between 60 mL/min and the 70–90 mL/min range did not achieve statistical significance, 70 mL/min represented the lowest tested irrigation rate that yielded a statistically significant reduction in extraction time compared to the 40 mL/min baseline. This suggests that targeting an irrigation flow rate of at least 70 mL/min during FURL with the SFV-UAS provides a reliable minimum threshold for consistently optimizing stone extraction efficiency.

The results of this experiment, which investigated the impacts of key intraoperative factors—intrarenal pressure, irrigation flow rate, and the diameter and length of the flexible access sheath—on stone extraction time, hold certain clinical significance. However, this study also has some limitations. First, the experiment was conducted in an *ex vivo* porcine renal model, lacking physiological variables such as active blood perfusion, continuous ureteral peristalsis, and respiratory movements. These physiological factors could significantly influence ureteral compliance and the hydrodynamics of stone evacuation. Second, the study utilized pre-fragmented human upper urinary tract stone fragments, which cannot fully simulate the continuous generation of irregular fragments and fine dust during actual laser lithotripsy, a factor that could pose a potential risk of sheath obstruction. In the future, it is necessary to conduct *in vivo* animal studies or prospective clinical trials combined with real-time IRP monitoring to further validate the safety and enhanced efficacy of the SFV-UAS.Furthermore, because the SFV-UAS and the conventional FV-UAS were manufactured by different companies, inherent variations in material composition, wall thickness, true internal diameter, and flexibility may exist despite identical nominal ureteral calibers (12/14 Fr). We acknowledge these proprietary hardware discrepancies as potential confounding factors that could independently influence hydrodynamic resistance and overall stone extraction efficiency. Third, the 420 extraction observations were derived from four *ex vivo* porcine kidney-ureter units, lacking complete biological independence. However, because stone evacuation via scope withdrawal is primarily a hydrodynamic process within a standardized access sheath, it is minimally affected by individual renal pelvic anatomy. With identical machine-controlled irrigation applied across all groups, inter-specimen anatomical variance was considered negligible, justifying our pooled statistical analysis. Fourth, the definitions of the stone extraction endpoint inherently differed between the two groups due to their structural designs. In the SFV-UAS group, the timing concluded when fragments reached the wedge-shaped junction, whereas in the FV-UAS group, it ended at the distal end of the sheath. Although we postulate these endpoints to be functionally comparable based on local hydrodynamics, their absolute equivalence was not directly validated in the present study. This methodological discrepancy warrants a cautious interpretation of the observed time differences.

## 5 Conclusion

Within the parameters of this *ex vivo* model,the novel SFV-UAS significantly enhances single-pass stone extraction efficiency during flexible ureteroscopy compared to FV-UAS. By effectively shortening the required mechanical withdrawal path, this innovative design effectively minimizes repetitive endoscopic manipulations, offering a promising and ergonomic clinical solution for managing large upper urinary tract stone burdens.

## Data Availability

The original contributions presented in the study are included in the article/Supplementary Material, further inquiries can be directed to the corresponding author.
